# Beyond benchmarking: Using WHO’s NPHA capability framework as a driver of institutional reform

**DOI:** 10.1371/journal.pgph.0006882

**Published:** 2026-07-17

**Authors:** Geoffrey Namara, Catherine Smallwood, Alexandra Zuber, Sara Hersey, Chikwe Ihekweazu

**Affiliations:** 1 Department of Health Emergency Intelligence & Surveillance, WHO Health Emergencies Programme, Berlin, Germany; 2 Ata Health Strategies, Washington, District of Columbia, United States of America; 3 WHO Health Emergencies Programme, Geneva, Switzerland; PLOS: Public Library of Science, UNITED STATES OF AMERICA

## Introduction

COVID-19 exposed weaknesses in national emergency preparedness and response (EPR) systems that were not primarily technical. Even countries with strong surveillance, laboratories, and emergency plans struggled to respond in time and at scale. An incoherent approach to governance also contributed, leading to fragmented authority, unclear accountability, and the absence of a single body empowered to coordinate across the emergency cycle [1,2].

Two landmark legal reforms have since reshaped global expectations: the 2024 amendments to the International Health Regulations (IHR) and the 2025 WHO Pandemic Agreement [2,3]. Both call for stronger national coordination and clearer institutional accountability but offer limited guidance on the institutional arrangements needed to deliver on these commitments. That gap is precisely what the WHO Framework for health emergency preparedness and response capabilities for national public health agencies (NPHAs) attempts to fill [4].

### A growing institutional landscape with uneven foundations

National public health agencies — also called national public health institutes, centres for disease control, or health security agencies — have expanded rapidly over the past two decades. By 2025, more than 120 countries had established or were establishing such institutions, up from fewer than 60 around the year 2000 [4].

Yet this quantitative growth masks profound institutional heterogeneity. In high-income countries, NPHAs often hold statutory authority, dedicated funding streams, and recognised scientific independence. In many low- and middle-income countries (LMICs), agencies operate with ambiguous legal mandates, dependence on external donors, limited workforce capacity, and uncertain relationships with ministries of health [5,6]. Policy debates have tended to emphasise institutional form — whether a country has an NPHA and what it looks like — while giving less attention to institutional function: the capabilities an agency requires to act effectively when an emergency strikes.

### The framework’s contribution: Defining capability alongside form

**Fig 1:**
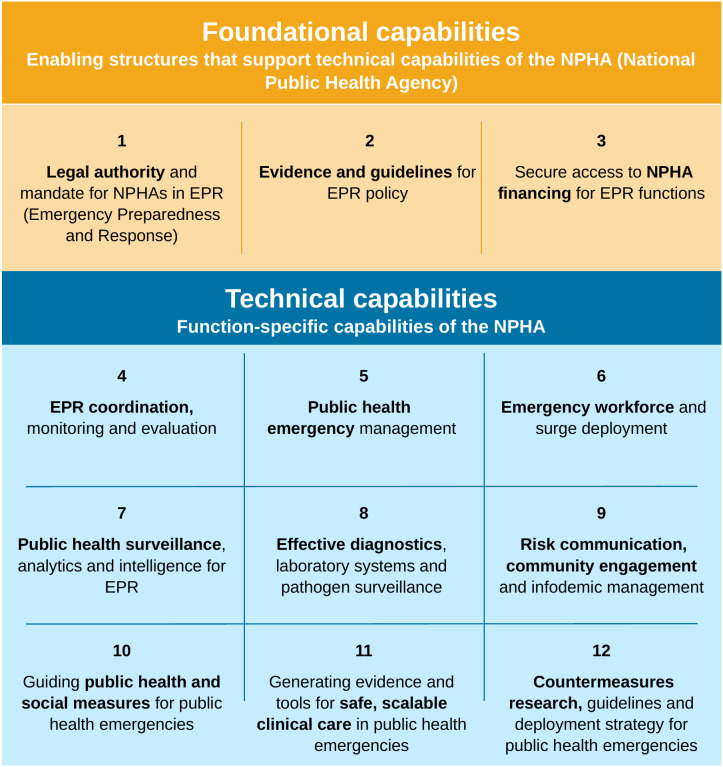
The 12 capabilities of the WHO Framework for EPR capabilities for national public health agencies.

The framework addresses both what NPHAs could look like and what they must be able to do. By defining 12 interdependent capabilities across foundational and technical domains, it provides a common reference point for strengthening agencies regardless of their organisational form (Fig 1). These span foundational domains — governance and legal authority, evidence generation and use for policy, and secure and flexible financing — and technical domains including surveillance and intelligence, laboratory and diagnostic systems, emergency coordination, workforce readiness, risk communication and community engagement, and access to countermeasures [4].

Central to the framework is the recognition that capabilities are interdependent. Foundational capabilities provide the enabling structures without which technical capabilities cannot function reliably, and technical capabilities reinforce each other across the emergency cycle. An NPHA without legally grounded emergency provisions cannot activate an incident management system when needed. Surveillance without confirmatory laboratory capacity generates uncertainty rather than intelligence. This systemic framing, rather than a checklist, encourages policymakers to invest across the capability set.

The framework acknowledges that NPHAs are not the only path to effective EPR governance. It describes four illustrative governance structures — from ministry-led systems to NPHA-led responses — recognising that institutional form must reflect national context. Indeed, institutional development carries real risks of cost and disruption if poorly designed. Nonetheless, the case for a dedicated institution such as an NPHA is structural: an institution that can generate evidence, communicate risk, and coordinate EPR functions under its own legal authority is better placed to provide accountability for EPR governance than a fragmented set of agencies with overlapping mandates. The framework is adaptable to decentralised systems and to countries at early stages of institutional development — precisely the contexts where the gap between existing capacity and what is needed is largest [6].

### Using the framework alongside existing tools

The NPHA framework does not replace existing EPR diagnostic tools — it builds on them. Joint External Evaluations (JEEs), State Party Self-Assessment Annual Reports (SPARs), and After-Action Reviews (AARs) identify where EPR gaps exist; the NPHA capability framework describes the institutional conditions required to address them. Used together, these tools can support a more coherent national investment cycle: diagnostic findings surface the gaps, the capability framework defines the institutional response, and legal and financing commitments turn definitions into action. The persistent challenge — that rich diagnostic data has not consistently translated into sustained investment [4,6] — is not a failure of the tools themselves but of the institutional and financing architecture around them.

Realising this potential requires that implementation be equity-sensitive and calibrated to country context. The framework is not intended to be applied as a uniform standard against which all countries are measured: LMICs face structural constraints — limited fiscal space, fragile health systems, and competing disease burdens — that cannot be resolved through technical assistance alone.

### Conclusion: A catalyst for EPR reform

The framework makes a timely and substantive contribution to global health security architecture. By defining what national public health agencies must be able to do, it shifts the policy conversation from institutional existence toward institutional function, and from gap identification toward the conditions required to close those gaps. Its value, however, depends on how it is used.

For NPHA leaders, the framework is a tool for internal assessment and external advocacy: to map capability gaps and to make the case for the investments required to translate those definitions into operational reality. For health ministers and finance ministries, it provides a structured basis for multi-year institutional investment — framed as the functional infrastructure that EPR governance requires. It helps reform-minded ministers identify functional gaps and duplications, and decide how to deliver EPR most effectively. For global health partners, it offers a common reference point for country support that is coherent, non-duplicative, and grounded in nationally defined needs.

Realising that value depends on treating the framework not as a benchmarking instrument but as a reform agenda — a basis for asking, in each country, whether the institutional arrangements in place are sufficient. NPHAs are central to that agenda where they exist, but the framework acknowledges that building effective EPR governance is incremental, context-dependent, and requires sustained political commitment across multiple institutions. What it offers is a precise, evidence-grounded description of functional, accountable EPR capability. For countries that want to close the gap between their preparedness plans and their response capacity, that clarity is where the work begins [6,7].
